# Spatiotemporal transcriptomic profiling reveals metabolic dysfunction prior to overt tauopathy in the PS19 mouse model

**DOI:** 10.21203/rs.3.rs-6941464/v1

**Published:** 2025-07-10

**Authors:** Shuai Wang, Moorthi Ponnusamy, Om Patel, Mitchell Hansen, Lisa Collier, Shane Collier, Gopal Thinakaran

**Affiliations:** 1 Byrd Alzheimer’s Center and Research Institute, University of South Florida, Tampa, FL, USA.; 2 Department of Molecular Medicine, USF Morsani College of Medicine, Tampa, FL, USA

**Keywords:** Alzheimer’s disease, frontotemporal dementia, spatial transcriptomics, tauopathy, metabolic dysfunction, Pgk1, glial activation

## Abstract

Abnormal accumulation of hyperphosphorylated tau in neurofibrillary tangles is a hallmark of neurodegenerative diseases, such as Alzheimer’s disease (AD) and frontotemporal dementia. In AD, tangle pathology characteristically develops in brain regions with heightened vulnerability, such as the entorhinal cortex and hippocampus. Emerging evidence implicates mitochondrial dysfunction and metabolic disturbances in AD progression, yet the relationship between regional vulnerability and pretangle tau-driven transcriptomic changes remains unclear. To address this critical gap, we utilized the tau P301S transgenic mouse model (PS19 line), which develops tau inclusions. Using spatial transcriptomic profiling across the hippocampal and cortical regions at selected disease stages, we captured spatiotemporal transcriptional responses to tauopathy. Our findings reveal that disease-associated microglia and astrocyte phenotypes emerge concurrently with phosphorylated tau accumulation across multiple brain regions. Intriguingly, the expression of *Pgk1*, a hub gene of the glycolytic pathway, was upregulated along with other metabolic pathway genes in the CA3 region at 2 months of age, preceding the onset of detectable tau tangle pathology, and correlated with tangle severity, suggesting early metabolic dysregulation in vulnerable regions. Further analysis of differentially expressed genes uncovered region-specific and temporally dynamic transcriptional patterns in the cortex and hippocampus. Early saturable alterations in ATP metabolic processes, glycolysis, and oxidative phosphorylation appeared in the hippocampus at two months of age, with delayed engagement in the cortical regions. These results underscore the contributions of metabolic stress and glial activation to tauopathy and regional vulnerability, highlighting spatial transcriptomics as a powerful tool for uncovering region-specific molecular insights into disease mechanisms.

## INTRODUCTION

Intraneuronal accumulation of filamentous tau is a key pathological feature of tauopathies. Primary tauopathies are caused directly by tau, whereas secondary tauopathies, such as Alzheimer’s disease (AD), occur when other factors trigger the tau pathogenic cascade^1–3^. Although filamentous tau is critical to several neurodegenerative diseases, the exact disease-specific mechanisms underlying neuronal dysfunction remain unclear. Multiple transgenic mouse models have been developed to study tau pathogenesis^4^. Among them, the PS19 line, which expresses FTDP-17-linked human P301S tau at approximately five-fold higher levels than endogenous mouse tau, has gained increased attention^5^. These mice exhibit a progressive accumulation of p-tau within neurons, leading to hippocampal synaptic abnormalities, microglial and astrocytic activation, followed by the formation of filamentous tau inclusions, culminating in neurodegeneration that results in lateral ventricular enlargement in advanced disease stages^5^. In patients with tauopathy, the progression of tau pathology typically manifests as spreading from the entorhinal cortex to the hippocampus and neocortex^6^; a similar regional vulnerability has also been observed in the PS19 mouse line.

As one of the body’s most energy-demanding organs^7^, the brain is highly sensitive to metabolic disturbances^8^. In the brains of individuals with AD, mitochondrial impairment is frequently observed before amyloid plaque pathology, resulting in reduced energy productionT^9–10^. Additionally, studies in mouse models have demonstrated that mitochondrial abnormalities are directly linked to the accumulation of β-amyloid and tau lesions, in the mouse brain^11–12^. Metabolic abnormalities accelerate neuronal damage and are closely associated with the gradual loss of cognitive function^13–14^. Proteomic profiling has revealed a correlation between neuronal and glycolytic signatures and t-tau levels in the cerebrospinal fluids from patients with AD^15^. Transcriptomic analyses have provided valuable insights into tau pathology-related disease mechanisms. However, our understanding of the early molecular changes associated with tauopathy remains incomplete. Moreover, the systematic molecular characterization of vulnerable brain regions at different disease stages before end-stage pathology presents a major challenge.

We applied a spatial transcriptomics approach to characterize the regional transcriptomic alterations and region-specific spatiotemporal changes across the hippocampal and cortical regions in PS19 tau transgenic mice at 2, 6, and 8 months of age. Our results reveal the earliest transcriptomic profiles in the hippocampus of 2-month-old PS19 mice before the manifestation of overt tau pathology. The expression of many metabolic pathway genes, including *Pgk1*, increased in the CA3 subregion prior to the onset of tau pathology and correlated with tau pathology severity, suggesting that early metabolic changes occur in vulnerable regions. The activation of genes regulating ATP metabolism peaks in the hippocampus at 2 months, followed by subsequent activation in cortical regions at later stages. Further investigation revealed regional transcriptomic differences in PS19 mice during the advanced stages of tau pathology. Thus, this study provides novel insights that enhance our molecular understanding of tauopathy.

## MATERIALS AND METHODS

### Ethics statement

All experimental animal procedures conformed to the guidelines approved by the University of South Florida’s Institutional Animal Care and Use Committee.

### Mice

*Emx1*-^IRES-*Cre*^ and PS19:*Emx1*-^IRES-*Cre*^ mice^16^ were maintained in the C57BL/6 background, and were used in this investigation as WT and PS19, respectively. The presence of the *Cre* allele is irrelevant to the focus of this study. Only male mice were used because of the sex differences in the extent of pathology in the PS19 line^5^.

### Tissue collection and processing

At 2, 6, and 8–9 months of age, the experimental mice were anesthetized using isoflurane and transcardially perfused with chilled 10 mM phosphate-buffered saline (pH 7.4; PBS). Subsequently, the brains were harvested and fixed in 4% paraformaldehyde and embedded in paraffin blocks. Five-μm thick sections were used for Digital Spatial Profiling (DSP) and immunostaining.

### Immunostaining

For DAB staining, tissue sections were processed as follows, using reagents and buffers purchased from Biocare Medical. First, the sections underwent antigen retrieval using the reveal decloaker RTU buffer in a Decloaking Chamber (Biocare Medical) for 30 min. This was followed by a 5-min incubation with a peroxidase blocking reagent, a 15-min incubation with Background Punisher before incubation with primary antibodies for 1 h at room temperature. Subsequently, the sections were incubated for 15 min with secondary antibodies, followed by a 20-min incubation with universal HRP tertiary reagent, and signal detection using a DAB^+^ chromogen kit. Finally, the nuclei were counterstained with hematoxylin and the slides were mounted with DPX mounting medium.

For immunofluorescence staining, the sections were incubated in Background Punisher (Biocare Medical) for 20 min, then primary antibodies for 1 h, followed by the respective secondary antibodies conjugated to Alexa Fluor (Life Technologies) for 1 h. Nuclei were stained with Hoechst, and the slides were mounted with Vectorshield aqueous antifade mounting medium (Vector Laboratories).

Stained sections were scanned on Olympus VS200 slide scanner with a 20 X objective and the images were analyzed using QuPath (v0.5.1-x64).

### RNAscope *in situ* hybridization

RNA *in situ* hybridization was performed using the RNAscope Multiplex Fluorescence Reagent kit v2 and probes from ACDbio, following the manufacturer’s instructions. Paraffin sections were baked at 60°C for 60 min and then deparaffinized in xylene. The sections were then treated with hydrogen peroxide for 10 min, followed by incubating in Target Retrieval Reagent at 94°C for 15 min. Subsequent steps included incubation with Protease Plus for 30 min at 40°C in a HybEZ II oven. Probe hybridization was carried out at 40°C for 2 h, followed by amplification steps. *Pgk1* RNAscope probe hybridization was optimized based on positive (*Polr2a, Ppib*, and *Ubc*) and negative (*DapB*) control probes. Finally, the sections were mounted with Vectorshield mounting medium and scanned as described above.

### GeoMX Whole Transcriptome Atlas (WTA) slide preparation

DSP experiments followed the manufacturer’s protocols (GeoMx DSP Slide prep manual: MAN-10115–04) with minor adjustments. Brain sections on slides were deparaffinized and processed through ethanol gradients before antigen retrieval at 100°C in Tris-EDTA buffer (pH 9.0) for 15 min under low pressure in a NESCO steamer. The slides were incubated with proteinase K (1 μg/ml) in PBS at 37°C for 15 min and then post-fixed in 10% neutral-buffered formalin for 5 min in a biosafety hood, followed by two 5-min washes in 0.1 M Tris Base, 0.1 M Glycine, and a final rinse in PBS. A 200 μl volume of diluted WTA probes in buffer R was added to each slide, covered with a Hybrislip (Grace Bio-Labs), and incubated for 15 h at 37°C in a HybEZ II oven humidified with 2x SSC buffer (0.3 M sodium chloride, 30 mM trisodium citrate, pH 7.0). The following day, Hybrislip covers were removed by rinsing briefly with 2x SSC containing 0.05% Tween-20. Slides were washed twice in 2x SSC containing 50% formamide for 25 min each and then twice in 2x SSC for 5 min each. Subsequently, the sections were stained with 1:10 dilution of SYTO 13 (Thermo Scientific) in Buffer W for 1 h. After staining, slides were washed twice in fresh 2x SSC, overlaid with Buffer S, and analyzed on the GeoMx DSP.

### GeoMX ROI Barcode Collection and Library Preparation

Sections were processed as described in the GeoMx DSP Library prep manual MAN-10117–04. Slides were imaged at 20x magnification, and SYTO13 nuclear staining was used to facilitate the selection of regions of interest (ROIs) and ensure at least 100 SYTO13-positive nuclei per region. Three ROIs were defined in the hippocampus (CA1, CA3, dentate gyrus [DG]), piriform cortex (PIR), retrosplenial cortex (RSP), and somatosensory cortex (SS) from each hemi-brain, yielding 12 ROIs per animal. ROIs were sequentially exposed to UV light (385 nm) one RO1 at a time, and the released barcodes were automatically aspirated from each ROI using a microcapillary system, and then deposited into individual wells of a 96-well plate to retain spatial resolution. The collected oligonucleotides were dried overnight and resuspended in 10 μl of DEPC-treated water. Library preparation was conducted using indexing PCR reactions. For each reaction, 4 μl of resuspended indexing oligonucleotides were combined with 4 μl of PCR primer pairs from the GeoMx Seq Code Pack Plate and 2 μl of NanoString 5x PCR Master Mix. Thermocycling conditions were as follows: 37°C for 30 min, 50°C for 10 min, and 95°C for 3 min, followed by 18 cycles of 95°C for 15 seconds, 65°C for 1 min, and 68°C for 30 seconds, with a final extension at 68°C for 5 min. PCR products were pooled and purified twice using AMPure XP beads (Beckman Coulter). The final pooled libraries were quantified with an Agilent 2100 Bioanalyzer and sequenced on an Illumina NextSeq 2000 platform using a 2×27 bp paired-end run with unique dual indexing with a 5% PhiX control spike-in at 400 pM.

### Sequencing data processing

Following sequencing, raw reads were processed using the GeoMx DND pipeline (v1, NanoString Technologies) according to the manufacturer’s protocol. First, the reads were trimmed, merged, and aligned to the reference list of indexing oligonucleotides to identify the corresponding source probes. Unique molecular identifiers (UMIs) were used to remove PCR duplicates, converting the processed reads into digital count conversion (DCC) files. DCC files were imported into the GeoMxWorkflows R for quality control and downstream analysis. Samples were required to meet quality thresholds of at least 10,000 reads and a minimum sequencing saturation of 50%. 190 samples passed these criteria and were included in subsequent analyses. Upper quartile (Q3) normalization was applied to ensure data quality further. The 75^th^ percentile of gene counts (the geometric mean of non-outlier probes per gene) was calculated for each ROI and normalized to the geometric mean of the 75^th^ percentile across all ROIs. Differential gene expression analysis was conducted using the DESeq2 R package, applying a negative binomial generalized linear model to 6,842 genes. This approach was used to identify differentially expressed genes (DEGs) within and across slides.

### Visualization of DEGs

The UpSet plot function from the R package ComplexHeatmap (v2.17.0)^17^ was used to compare the trend of DEGs along tau pathology development. The ggplot2 package was employed to create volcano plots for visualizing DEGs. Gene ontology (GO) analysis of DEGs was performed and visualized using the R package clusterProfiler [v4.8.2]^18^. The significance levels for GO terms and the KEGG pathway were set at *p* < 0.05. To identify dynamic expressed genes (DDEGs) during aging, the R package TrendCatcher^19^ (v1.0.0) was used with the thresholds logFC.thres = 0.5, and dyn.gene.p.thres = 0.05.

### Gene expression signature scores and correlation with tau staining intensity

To score a gene expression dataset against a gene set, we used the R package singscore (v1.21.0)^20^. For the neurodegenerative disease-associated microglial (DAM) signature score, 271 DEGs between the stage 2 DAM cluster and the homeostatic cluster from a scRNA-seq study^21^ were retained with the cutoff log2|fold change|>0.3, resulting in 113 genes overlapping with our dataset. We used DEGs between the disease-associated astrocyte (DAA) Clusters 4 and 1 from a scRNA-seq study^22^ for the DAA signature score; 254 genes were retained with the cutoff log2|fold change|>0.3, leading to 163 overlapping genes in our dataset. The molecular signatures underlying the neurofibrillary tangle (NFT) were calculated against an AT8^+^ sorted neuron’s snRNAseq data^23^; 257 genes were retained with the cutoff log2|foldchange|>0.3, resulting in 247 overlapping genes in our data set. The AT8^+^ intensity values from the DAB staining images were used to perform Spearman correlation with all mRNA counts read from the same animal and region. The correlation coefficient and −Log_10_(*p*-value) were then plotted as a volcano plot.

### Statistical Analysis

R (v4.4.1) was used for all data analyses. Two-tailed unpaired *t*-tests were employed to compare two groups. Two-way ANOVA was used to determine the differences between age and genotype in analyzing gene set signatures. A negative binomial generalized linear model in DESeq2 was utilized to model RNA-seq count data, and the Wald test was performed to call the DEGs, followed by the Benjamini-Hochberg procedure to adjust p-values for multiple testing. In the volcano plots, the x-axis values represent gene expression levels in PS19 compared to WT animals. All values are presented as mean ± SEM in Fig. 4A and Fig. 5D. The significance levels are defined as follows: *p < 0.05, **p < 0.01, ***p < 0.001, and NS (*p* > 0.05).

## Results

### Spatial transcriptomics captures signatures from selected mouse brain regions.

**Figure 1:**
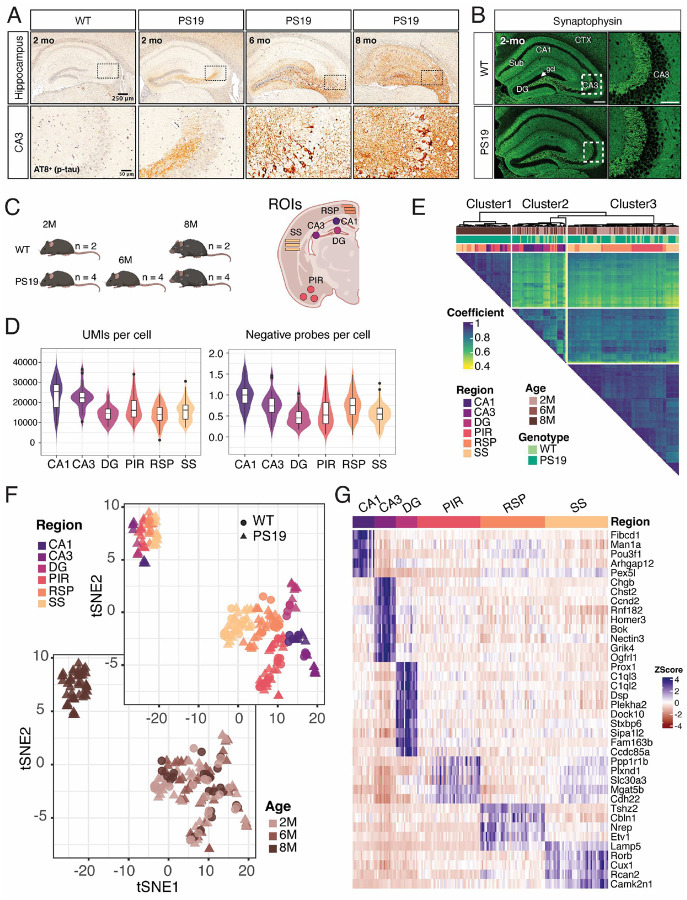
Spatiotemporal transcriptomic profiling of the PS19 tauopathy model. **(A)** Immunohistochemical analysis of p-tau (pSer202/pThr205) levels in the hippocampus and CA3 region of male PS19 mice at 2, 6, and 8 months of age, analyzed using mAb AT8 staining. **(B)** Immunofluorescence images show Synaptophysin expression in the hippocampus of 2-month-old WT and PS19 mice. **(C)** Sampling from WT and PS19 mice at three stages of tau pathology. Regions of interest (ROIs) included hippocampal subfields CA1, CA3, DG, and cortical regions PIR, RSP, and SS. **(D)** Violin and box plots display the Unique Molecular Identifiers (UMIs) and negative probes per cell for each indicated region. **(E)** The Pearson correlation for 6842 genes across 190 ROIs is represented as a heatmap. The ROIs were grouped into three clusters: Cluster 1: 8-month severe tau pathology cluster; Cluster 2: Non-pathology hippocampus cluster; Cluster 3: Non-pathology cortex cluster. The coefficient is color-coded, with darker colors indicating a higher Pearson correlation coefficient. The three annotation rows at the top are color-coded for age, genotype, and region, respectively. (**F**) t-distributed stochastic neighbor embedding (tSNE) plot of all ROIs identified two major transcriptionally distinct clusters. **(G)** Gene expression levels of spatial markers for six selected regions. Color intensity represents the marker’s expression levels in each ROI.

In PS19 mice, mAb AT8^+^ phosphorylated tau appears in the mossy fibers of the hippocampus of as early as two months of age; no AT8 staining is observed in WT mice (Fig. 1A). The staining becomes more intense by 6 months, with visible accumulation of p-tau in CA3 neurons in addition to the neuropil in the DG and CA3 areas. By 8 months, the entire hippocampus shows intense AT8 staining and intraneuronal p-tau accumulation, accompanied by noticeable overall shrinkage (Fig. 1A). In the cortex, AT8^+^ p-tau staining was observed in the retrosplenial, somatosensory, and piriform cortex areas at 2 months of age, which became more intense at 8 months (Supplementary Fig. 1).

To understand the regional response to tau P301S expression, we conducted spatial transcriptomics analysis of PS19 mice at the ages of 2, 6, and 8 months, corresponding to mild, moderate, and severe tau pathology, respectively, using the GeoMx DSP platform. Age-matched non-transgenic mice (WT) at 2 and 8 months were also included to delineate pathology-associated *versus* age-associated transcriptional changes. We focused our attention on six ROIs: CA1, CA3, and DG from the hippocampus and RSP, SS, and PIR from the cortex (Fig. 1C). Analogous to 10x Visium and other single-cell transcriptomics approaches, our spatial transcriptomic dataset revealed 15,000 to 30,000 unique reads per cell (Fig. 1D). The number of negative probes detected in each cell was found to be lower than 1 (Fig. 1D). A hierarchically clustered triangle heatmap was generated based on Pearson’s correlation analysis of Q3 normalized expression levels of all 6,842 genes detected from 190 ROIs after ROI and probe QC filtering (Fig. 1E). The correlation heatmap shows three different groups: Cluster 1 denotes the ROIs from the hippocampus and cortex with severe PS19 pathology, Cluster 2 denotes the hippocampus, and Cluster 3 includes most regions from other cortical areas without severe pathology. The t-distributed stochastic neighbor embedding (tSNE) plots of 190 ROIs from different brain regions show transcriptionally distinct clusters, with the 8-month-old PS19 cohort separating from the others (Fig. 1F). The expression of spatial transcriptional markers, identified by the FindAllMarkers function in the Seurat package^24^ and displayed as a heatmap, confirms the veracity of our DSP sampling (Fig. 1G).

### NFT-associated transcriptomic signature in the hippocampus of P301S tau mice before overt tau pathology

**Figure 2:**
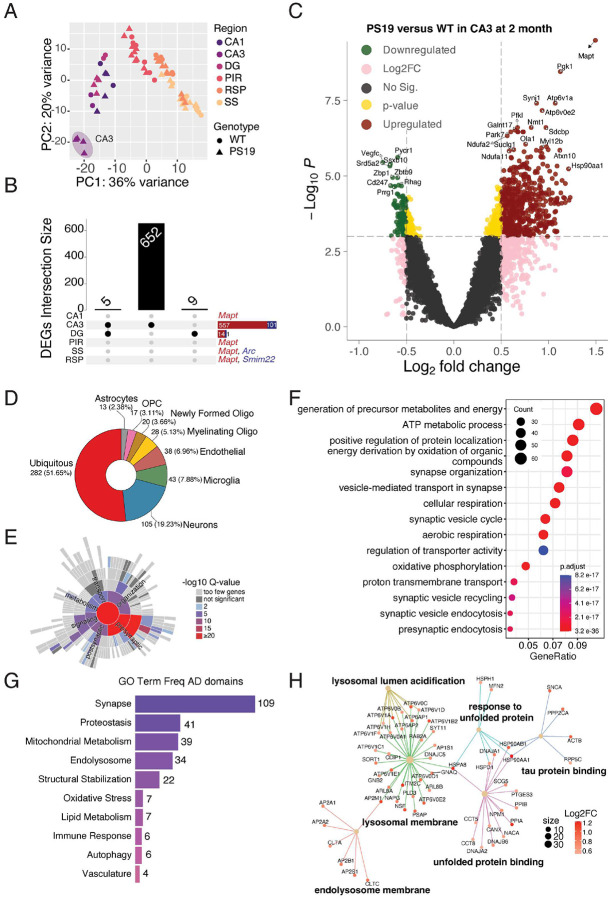
Spatial transcriptomic signatures of PS19 mice at 2 months of age. **(A)** The results of PCA on 2-month-old ROIs show that the first two principal components account for the specified percentages of data variation along the x- and y-axes, respectively. **(B)** The UpSet plot illustrates the DEGs from all six regions, with counts for CA1, CA3, DG, PIR, RSP, and SS being 1, 658, 15, 1, 2, and 2, respectively. **(C)** The volcano plot depicts the DEGs between PS19 and WT mice in the CA3 region at 2 months of age, with color-coded symbols indicating the fold change and *p* values relative to the thresholds. Green: *p* < 0.01, and log2 (fold change) < −0.5; Red: *p* < 0.01, and log2 (fold change) > 0.5; Pink: *p* > 0.01, and |log2 (fold change) | > 1; Gold: *p* < 0.01, and |log2 (fold change) | < 0.5; Grey: *p *> 0.01, and | log2 (fold change) | < 0.5. **(D)** Donut chart showing cell type annotation on 658 DEGs. **(E)** SYNGO (Synaptic Gene Ontologies) Biological Process terms from the 658 DEGs in CA3 are depicted as a sunburst plot, with colors indicating the significance levels of the GO domains. (**F**) The GO Biological Process analysis on all 658 DEGs, with the gene count for each GO term represented by the circle size, where red and blue colors denote the adjusted *p* values. **(G)** Disease-specific GO terms derived from AD-associated GO terms defined by the TREAT-AD Center at Emory-Sage-SGC are categorized into ten major domains, with numbers indicating the terms belonging to each domain. **(H)** The network visualization of tau protein binding, unfolded protein-related, and lysosome-related GO terms from the gene enrichment analysis results, depicted in **G**, with colors representing the log2(fold change) of the DEGs.

Principal component analysis (PCA) of 2-month-old PS19 and WT mice revealed that the first two principal components (PC1 and PC2) collectively explained 56% of the variance in the dataset. PC1 primarily accounted for the regional differences (36% variance), which were largely independent of genotype (Fig. 2A). The PCA plot also showed a clear separation of the PS19 CA3 region from other areas of PS19 and WT mice, indicating a significant pathology-specific difference in the PS19 transcriptome within this hippocampal subregion at 2-months of age, prior to the manifestation of overt tau pathology (Fig. 2A). At 2-months of age, synaptophysin staining in the CA3 region remained unaffected (Fig. 1B). By applying cutoffs of *p*<0.001 and |log2(fold change)| > 0.5, we identified 1, 658, 15, 1, 2, and 2 DEGs in the CA1, CA3, DG, PIR, SS, and RSP, ROIs respectively (Fig. 2B). The most surprising finding was a large number of DEGs in the CA3 region (658 total; 557 up- and 101 down-regulated), with 652 being unique to the CA3 region (Fig. 2B and C). The Volcano plot shows the up- and down-regulated DEGs in the CA3 (Fig. 2C) and DG (Supplementary Fig. 3G) regions. In addition to the expected higher levels of transgene-derived human and endogenous mouse tau transcripts, collectively referred to as *Mapt*, genes including *Pgk1*, *Synj1*, *Atp6v1a*, and *Atp6v0e2* were significantly upregulated. Transcripts corresponding to *Pycr1, Vegfc, Ssxb10, and Srd5a2* were downregulated in the PS19 mouse CA3 region (Fig. 2C).

We used the RNAseq data from purified cell types^25^ to generate cell-type annotations of the DEGs in the CA3 region of PS19 mice. The results revealed that 282 DEGs were expressed in more than one cell type (51.65%), with 105 expressed uniquely in neurons (19.23%), 43 in microglia (7.88%), 38 in endothelial cells (6.96%), 28 in oligodendrocytes (5.13%), 20 in newly formed oligodendrocytes (3.66%), 17 in oligodendrocyte precursor cells (3.11%), and 13 in astrocytes (2.38%) (Fig. 2D). Further Synaptic Gene Ontology analysis of the CA3 DEGs revealed that a greater proportion of the biological process and cellular component terms ((Fig. 2E and Supplementary Fig. 3D and E) were associated with synaptic vesicles and presynaptic sites. To elucidate the enriched biological processes across all cell types, we performed Gene Ontology (GO) analysis on all 658 DEGs, as well as on the upregulated and downregulated DEGs separately. The top 15 functional annotations for all DEGs are shown as dot plots (Fig. 2F). Additionally, the top 10 annotations for upregulated and downregulated DEGs are shown as dot plots separately (Supplementary Fig. 3A and B). The GO results identified pathways in the lysosomal membrane, synaptic vesicle membrane, endocytosis, aerobic respiration, translation, and membrane rafts. The genes associated with the top three enriched biological process terms from the upregulated DEGs in CA3 are depicted as a circular network (Supplementary Fig. 3C). An application of human brain AD-specific GO terms defined by the TREAT-AD Center at Emory-Sage-SGC^26^ to our dataset highlights similar transcriptomic changes in the synapse, proteostasis, mitochondrial metabolism, endosome, and structural stabilization (Fig. 2G and Supplementary Fig. 3F). The enriched pathways representing tau protein binding, unfolded protein binding, response to unfolded proteins, lysosomal membrane, and endolysosome membrane in the CA3 region from PS19 mice are displayed as a gene network (Fig. 2H). At the center of the gene network, *Hsp90ab1* and *Hsp90aa1* are chaperones that bind to pathological tau and coordinate protein and synaptic homeostasis^27–28^. The upregulation of *Hsp90ab1* and *Hsp90aa1* indicates the cellular response to accumulated pathological tau species.

### *Pgk1* expression is upregulated in the CA3 region and serves as a hub gene for energy metabolism.

**Figure 3:**
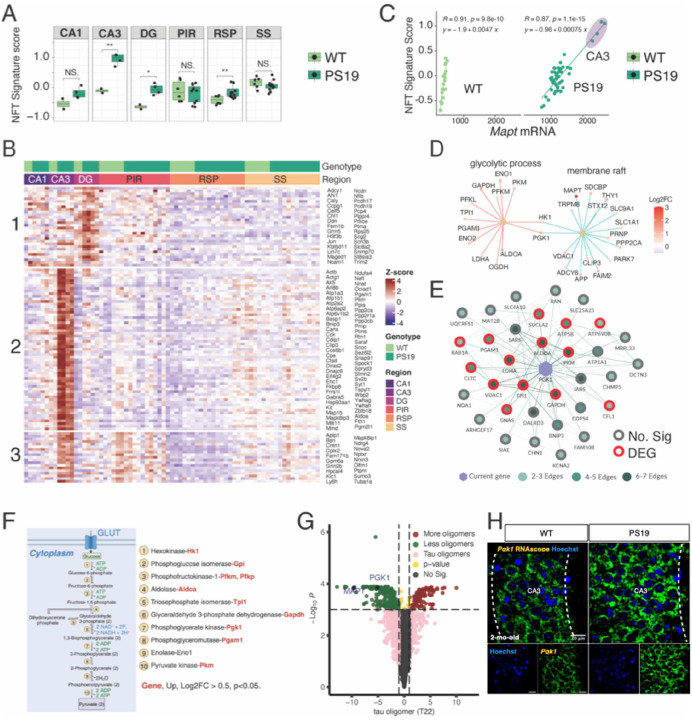
Tau-driven spatial transcriptomic signatures precede overt tau accumulation. **(A)** NFT signature scores of genes from a snRNA dataset of AT8^+^ cells from patients with AD in the regions analyzed in our study. Two-tailed and unpaired t-test. **(B)** A heatmap depicting the expression levels of the NFT gene set. The NFT genes are grouped into three clusters. Cluster 1: DG-specific NFT gene set; Cluster 2: CA3-specific NFT gene set; Cluster 3: Shared NFT gene set. **(C)** The correlation between *Mapt* mRNA levels and NFT signature scores in WT (light green dots) and PS19 (dark green dots) ROIs. **(D)** *Pgk1* gene network visualization of the gene enrichment analysis results. The color indicates the log2(fold change) of each DEG. **(E)** *Pgk1* co-expressed genes based on a co-expression network analysis of RNA-seq data from AD cases and controls (Agora). Each node represents a different gene. The green shade of the circles indicates the frequency of significant co-expression; the red outline of the circles indicates that the gene is among the DEGs in our dataset. **(F)** Protein-coding genes for glycolytic enzymes in the glycolysis metabolic pathway are illustrated by BioRender. **(G)** Volcano plot of hit genes from CRISPR screens in the iPSC-derived neuron. Positive hits are in red, and negative in green. *MAPT* and *PGK1* are among the top negative hits. **(H)** RNAscope staining results for *Pgk1* in the CA3 region of 2-month-old WT and PS19 mice.

The presence of NFTs in the neocortex, amygdala, hippocampus, and brain stem has been reported in PS19 mice at six months of age^5^. We investigated whether pretangle pathogenic tau-associated transcriptomic signatures emerge in 2-month-old PS19 mice. Using a snRNAseq dataset of AT8^+^ cells from patients with AD^23^, we observed significantly higher NFT signature scores in the transcriptomes of the CA3, DG, and RSP regions of PS19 mice even at 2 months of age (Fig. 3A). The gene set for the NFT signature in our dataset is displayed as a hierarchically clustered heatmap. The largest cluster was a CA3 unique gene set (Fig. 3B). The NFT signature score is tightly correlated with *Mapt* levels (Fig. 3C) in the ROIs from PS19 mice (R = 0.87, *p* = 1.1×10^−15^) and WT mice (R = 0.91, *p* = 9.8×10^−10^), accurately reflecting the molecular signatures driven by mutant human tau P301S expression.

A proteomic study of CSF from individuals with AD found a correlation between glycolytic signature, which includes PGK1 and other glycolytic enzymes, and tau levels^15^. The *Pgk1* gene is the second-most upregulated gene in CA3, surpassed only by *Mapt*, in 2-month-old PS19 mice. *Pgk1* is classified under the GO terms glycolytic process and membrane raft (Fig. 3D). We obtained a co-expression network for PGK1 based on RNA-seq data from AD cases and controls from the Agora database^26^, which hosts high-dimensional human transcriptomic, proteomic, and metabolomic evidence regarding gene associations with AD. Overlaying our RNAseq results indicated that the *Pgk1* gene could be a potential hub gene for the glycolytic process (Fig. 3E). Upon further inspection, we observed that all brain-expressed genes encoding glycolysis pathway enzymes were upregulated in the CA3 region of PS19 mice at 2 months of age (Fig. 3F). Interestingly, a recent genome-wide CRISPRi-based modifier screen^29^ revealed that the knockout of *PGK1* results in fewer tau oligomers in human iPSC-derived neurons (Fig. 3G). This data from an unbiased screen aligns with our findings of elevated *Pgk1* transcripts in CA3, concurrent with the accrual of transgene-derived P301S as AT8^+^ p-tau (Fig. 1A). We validated the elevated *Pgk1* mRNA in the CA3 region of 2-month-old PS19 mice by performing RNAscope *in situ* hybridization (Fig. 3H). Our observation is also consistent with prior research suggesting that early PGK1 activation in the cytosol may disperse pathogenic protein aggregates by increasing ATP levels and clearing these proteins via autophagy^30^. These findings indicate that the glycolytic process, a highly conserved and fundamental pathway in all species, is upregulated as one of the earliest compensatory responses to higher p-tau levels in the hippocampal CA3 region.

### Regional transcriptomic signatures of P301S tau mice at the late tau pathology stage.

**Figure 4:**
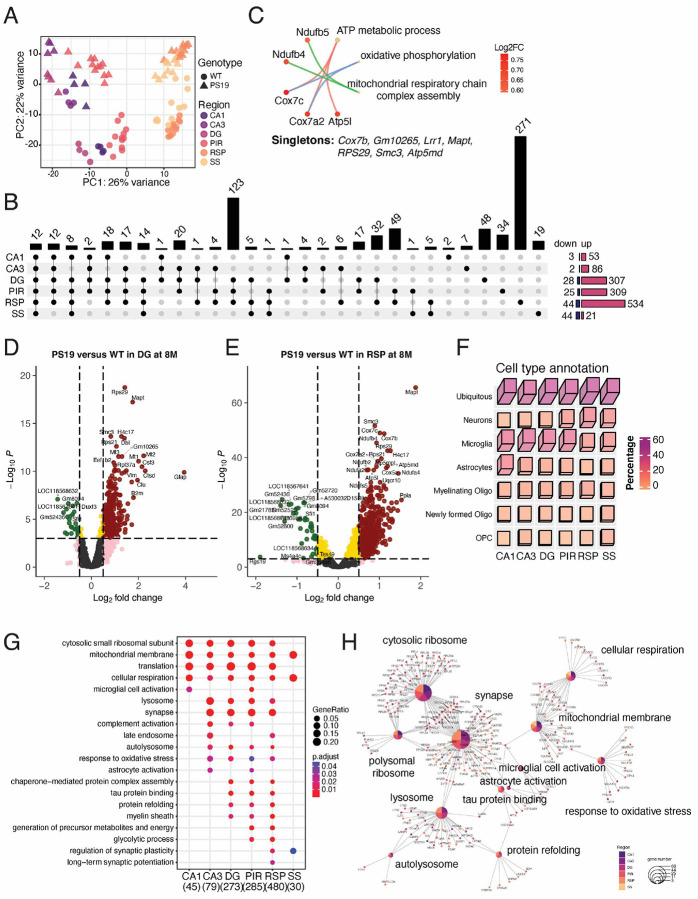
Spatial transcriptomic profiling of PS19 mice at the severe stage of tau pathology. **(A)** The PCA results of 8-month-old ROIs. The first two principal components explain the indicated percentages of data variation on the x- and y-axes, respectively. **(B)** The UpSet plot illustrates the number of DEGs from all six regions in 8-month-old PS19 mice compared to age-matched WT animals. The DEGs for CA1, CA3, DG, PIR, RSP, and SS are 56, 88, 335, 334, 578, and 63, respectively. **(C)** Results of GO analysis for the 12 DEGs shared by all six brain regions. (**D and E**) Volcano plots depicting the DEGs between PS19 and WT in the DG **(D)** and RSP **(E)** regions at 8 months of age. Color-coded symbols indicate the fold change and *p*-values, showing whether they are below or above the thresholds. Green: *p* < 0.01, and log2 (fold change) < −0.5; Red: *p* < 0.01, and log2 (fold change) > 0.5; Pink: *p* > 0.01, and |log2 (fold change) | > 1; Gold: *p* < 0.01, and |log2 (fold change) | < 0.5; Grey: *p* > 0.01, and | log2 (fold change) | < 0.5. **(F)** A 3D heatmap depicts the percentage of cell types for the DEGs in six regions, with height and colors indicating the ratio of cell types. **(G)** The dot heatmap represents the results of the GO analysis for the DEGs from all six regions of 8-month-old mice, with the gene ratio in each GO term represented by the circle. Red and blue colors indicate the *p*-values. **(H)** Gene network visualization of the gene enrichment analysis results. The colors of the center node indicate the regions in which the enriched pathway terms appear, while the node size reflects the number of genes associated with the term.

We further investigated the transcriptomics of select brain regions in 8-month-old PS19 mice, comparing them to age-matched WT mice. PCA showed that brain regions and genotypes contributed to the observed variance on PC1 (26%) and PC2 (22%) in this transcriptomic dataset (Fig. 4A). Thus, unlike the CA3-limited DEGs at 2-months of age, P301S tau expression significantly affected gene expression at 8-months of age in all brain regions examined in this study. An UpSet plot was constructed to visualize DEGs across the ROIs: 56 in CA1 (53 up- and 3 down-regulated), 88 in CA3 (86 up- and 2 down-regulated), 335 in DG (307 up- and 28 down-regulated), 334 in PIR (309 upregulated and 25 downregulated), 578 in RSP (534 upregulated and 44 downregulated), and 65 in SS (21 upregulated and 44 downregulated) (Fig. 4B). The relatively fewer DEGs observed in the CA1 and CA3 regions (Fig. 4B and Supplementary Fig. 4A and B) likely results from tauopathy-induced neuronal loss at this late stage of pathogenesis in PS19 mice (Fig. 1A)^5^. In addition, only a few DEGs (all 12 upregulated) were common to all six analyzed regions; the products of these 12 genes participate in biological processes such as ATP metabolic process, oxidative phosphorylation, and mitochondrial respiratory chain complex assembly (Fig. 4C). The DEGs from the DG region included markers of glial cell activation, such as *B2m*, *Clu*, *Cst3*, *Ctsd*, *Vim*, and *Gfap* (Fig. 4D). Although fewer DEGs were observed in the CA1 and CA3 regions compared to the DG area (Fig. 4B and Supplementary Fig. 4A and B), the percentages of microglial genes are comparable in all hippocampus regions (Fig. 4F). Moreover, the DEGs from the RSP region are involved in various biological processes, and genes such as *Cox7b*, *Cox7c*, *Ndufb4*, and *Ndufa4* encode the mitochondrial membrane proteins (Fig. 4E). Similar, but fewer genes were identified as DEGs in the PIR and SS cortex (Supplementary Fig. 4C and D). The DEGs across the six regions also revealed varied cell type representations (Fig. 4F). At the late tau pathology stage, a relatively higher proportion of microglial and astrocytic DEGs were found in the CA1, CA3, DG, and PIR regions. In contrast, fewer glial-specific DEGs and a greater number of neuronal DEGs were identified in the RSP and SS regions of the cortex (Fig. 4F). The Gene Ontology analysis of the DEGs from the ROIs, compared against manually curated AD-relevant biological terms^26^, captured region-dependent and region-independent signatures (Fig. 4G and H). The enriched GO terms, such as mitochondrial membrane and cellular respiration, were identified in all regions. Following the abnormal glycolytic process in the early stage of CA3 (at 2 months), the cortical regions displayed a similar modulation of the glycolytic process during the late tau pathology stage (8 months) (Fig. 4G).

### Progressive glial activation in the hippocampus and cortex of P301S tau mice.

**Figure 5:**
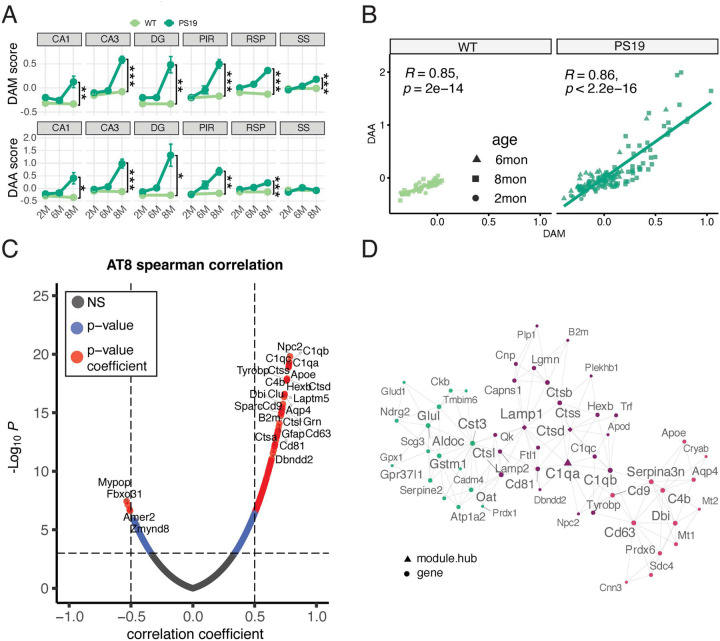
Progressive glial activation in the hippocampus and cortex of P301S tau mice. **(A)** DAA and DAM signature scores for six brain regions at three stages. Two-way ANOVA was used to determine the differences between ages and genotypes. Only the differences between genotypes are indicated. **(B)** The correlation between DAA and DAM signature scores in WT (light green dots) and PS19 (dark green dots) ROIs. **(C)** The Spearman correlation between AT8 intensity and mRNA levels for all ROIs. **(D)** *C1qa* was identified as a hub gene for DEGs in the 8-month-old PS19 hippocampus by MEGENA.

To further characterize the subtypes of regional glial cell activation in response to accumulating tau pathology, we interrogated our spatiotemporal transcriptomic dataset against microglia and astrocyte subtypes defined by snRNA transcriptomic analysis in various AD models, such as disease-associated microglia (DAM)^21^ and disease-associated astrocytes (DAA)^22^. We detected increased DAM and DAA signature scores correlating with the development of tauopathy in the analyzed brain regions (Fig. 5A). For the DAM signature score, all six regions exhibited a similar pattern, with DAM marker expression beginning to increase as early as six months of age. The DAM signature scores for the CA3, DG, and PIR regions were comparable and higher than those for the CA1, RSP, and SS regions. With respect to the DAA signature score, more than half of the brain regions displayed similar upregulation patterns to those of the DAM signature scores, except for the RSP and SS cortex, where little to no activation was observed. The overall correlation between the DAA and DAM signature scores indicates that tauopathy activates the microglia and astrocytes at the same age and to a similar extent in the PS19 model in the hippocampus and piriform cortex (Fig. 5B).

As transcriptomic signatures reflect tauopathy-driven molecular perturbations, the relative regional differences in transcript levels between PS19 and WT mice likely stem from regional variations in the severity of tau pathology. To confirm this notion, correlations between all mRNA levels and the three severity stages of tau pathology, defined by the AT8^+^ tangle density, were calculated and illustrated as volcano plots (Fig. 5C). Notably, 117 genes, including *C1qa*, *C1qb*, *C1qc*, *Npc2*, *Ctss*, *Tyrobp*, and *Apoe*, exhibited strong correlations with AT8 intensity in 8-month-old animals. The Multiscale Embedded Gene co-Expression Network Analysis (MEGENA)^31^ identified clusters enriched in neuroinflammatory pathway genes (Fig. 5D) and the cluster’s hub gene, *C1qa*, which is significantly associated with AD amyloid and tangle pathology^32–33^ as well as complement-mediated synaptic loss^34^.

In addition to comparing PS19 and WT mice at 8 months of age, we performed DEG analysis by comparing 2- and 8-month-old PS19 mice (Supplementary Fig. 5A) and 2- and 8-month-old WT mice (Supplementary Fig. 5B). The aging PS19 mice exhibited significantly more DEGs in every region profiled in this study (Supplementary Fig. 5C). A notable difference in the number of upregulated and downregulated DEGs was observed between the hippocampus and cortex of PS19 mice (Supplementary Fig. 5C). A greater proportion of downregulated DEGs was identified in the hippocampal subfields; however, more upregulated DEGs were found in the cortical regions analyzed. Only a few DEGs were shared between the 2-month vs. 8-month comparisons in WT and PS19 mice in any region analyzed. Older PS19 mice displayed differential expression of genes corresponding to cytosolic small ribosomal subunits and synapse function in nearly all explored regions (Supplementary Fig. 5D and F). Hippocampus-specific enriched biological processes included microglial cell activation and astrocyte activation; conversely, the cortex-specific enriched biological processes encompassed cellular respiration and the mitochondrial membrane (Supplementary Fig. 5D and F). The extent of differential gene expression between 2-month vs. 8-month WT mice is particularly notable in the RSP and SS regions (Supplementary Fig. 5E and G). Together, these results reveal progressive glial activation in the hippocampus and cortex of PS19 mice, marked by an elevated gene set centered on *C1qa*, resulting from a surge of AT8^+^ tau pathology tangles at 8 months of age.

### Spatiotemporal transcriptomic signatures in P301S mice reveal regional disease progression.

**Figure 6:**
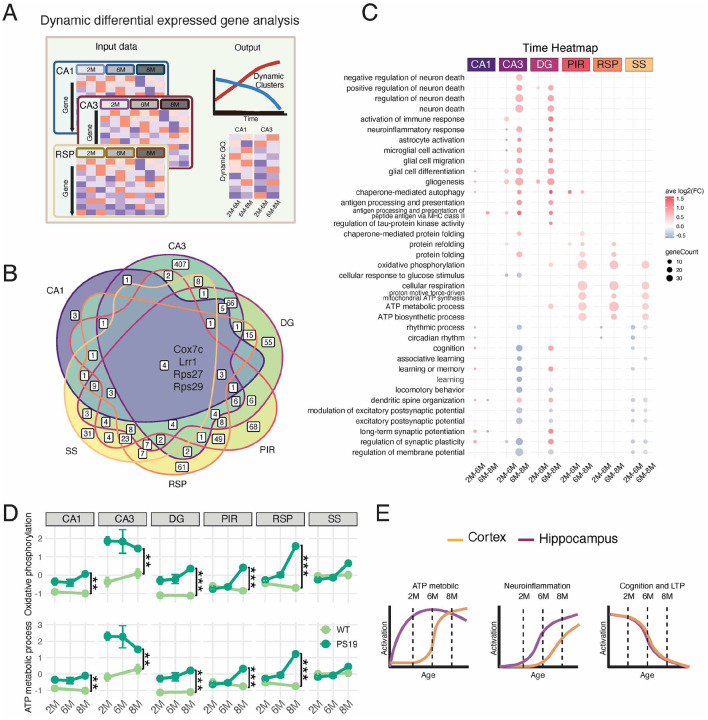
Spatiotemporal transcriptomic signatures in P301S reveal regional disease progression. **(A)** Illustration of TrendCatcher data processing and GO TimeHeatmap outputs. **(B)** Venn diagram of DDEGs in six brain regions. **(C)** Integrated GO TimeHeatmap for six brain regions, where the dot color indicates the average log2(fold change) of genes in the enriched pathway term, and the dot size represents the number of genes associated with that term. **(D)** ATP metabolic process and oxidative phosphorylation signature scores for six brain regions at three stages. Two-way ANOVA was used to determine the differences between ages and genotypes; only the differences between genotypes are indicated. **(E)** Summary of the dynamic regulation of ATP metabolic processes, neuroinflammation, cognition, and LTP in the cortex and hippocampus ROIs during tau pathology development in PS19 mice. The curves were generated in BioRender.

In addition to the commonly used methods for identifying DEGs between two groups, we employed a novel approach to identify dynamic differentially expressed genes (DDEGs). In this approach, schematically represented in Fig. 6A, we utilized TrendCatcher^19^ to identify DDEGs in 2-, 6-, and 8-month-old PS19 mice within each selected region. The intersection of DDEGs among brain regions is summarized in a Venn diagram (Fig. 6B). Only 4 DDEGs were identified in all analyzed brain regions. This small portion of shared DDEGs is akin to the limited number of shared DEGs depicted as Upset plots generated from spatial transcriptomics data of PS19 mice at 2 months (Fig. 2B) and 8 months of age (Fig. 4B). These findings underscore regional heterogeneity and the cellular environment critical for cellular activities and transcriptional responses to pathogenesis, thereby emphasizing the necessity of spatial transcriptomic and proteomic profiling. The gene ontology analysis of DDEGs for each brain region was visualized in an enriched GO TimeHeatmap (Fig. 6C). In accordance with the published data^5^, our results show that neuronal death and glial cell activation begin in the hippocampal regions, particularly in the CA3 and DG, after 6 months of age. However, increased glial cell differentiation and gliogenesis were observed at an earlier age in the CA3 and DG regions. Intriguingly, our analysis reveals that the hippocampus of PS19 mice exhibited saturable enrichment of the ATP metabolic process, oxidative phosphorylation, and protein folding pathways as early as 2 months of age, with minimal dynamic regulation during late pathogenic stages (Fig. 2F, Fig. 6D). In contrast, in the cortical regions analyzed in this study, the aforementioned pathways are activated between six and eight months of age, reaching a similar maximal activation level observed in the CA3 region (Fig. 6D). Our spatial transcriptomic dataset also detected region-independent signatures, such as decreased gene expression related to circadian rhythmic processes, cognition, and modulation of excitatory postsynaptic potential. These region-independent signatures could provide transcriptomic evidence for disrupted sleep^35^ and memory decline^36^ in PS19 mice at 6 months or even earlier. The trends of region-dependent and region-independent spatiotemporal transcriptomic signatures are schematically summarized in Fig. 6E.

## DISCUSSION

This study employed a spatial transcriptomic approach to investigate the early and progressive molecular changes in the PS19 tauopathy model. Unlike previous studies that utilized bulk tissue from aged animals^37,39^, this study sought to identify transcriptomic changes prior to neurodegeneration. We examined the hippocampal and cortical subregions at 2, 6, and 8 months to identify region-specific transcriptomic signatures that correlate with tau pathology. Our results indeed reveal that *Mapt/MAPT* mRNA levels in PS19 are significantly higher in the CA3 region relative to other brain regions analyzed (Supplementary Fig. 2), although AT8^+^ p-tau staining was visible in all regions analyzed. The DSP approach allowed us to map transcriptomic changes in select regions across early and late disease stages, highlighting previously unrecognized metabolic and synaptic alterations associated with the progression of tau pathology. Furthermore, by systematically characterizing hippocampal and cortical regions over time, we reveal that CA3 is a uniquely vulnerable area with distinct transcriptomic changes, in contrast to earlier studies that focused on the entire hippocampus or cortex. A key finding of this study is the identification of early transcriptome alterations in the CA3 subregion of the hippocampus, which precede overt tau pathology. Notably, *Pgk1*, which encodes an essential glycolytic enzyme, is upregulated in the CA3 region as an early metabolic response to pathological tau accumulation. By correlating our findings with published human CSF proteomic and iPSC-derived neuronal studies, we provide translational relevance to AD pathogenesis, suggesting *Pgk1* upregulation as an early marker of tau pathology.

Spatial transcriptomic approaches coupled with next-generation sequencing have been developed over the last decade^40^ and are widely used in molecular biology studies to obtain unbiased insights into regions of interest at the whole transcriptome level. The compatibility of the GeoMX WTA method with FFPE tissue sections makes it suitable for discovery research in samples from fixed tissue collected over time. However, this approach lacks the spatial resolution needed to capture transcriptome signatures from individual cells. By leveraging publicly available scRNA-seq and snRNA-seq data from patients with AD and disease models^21,23^, we applied cell annotation to our dataset to address the spatial limitations of the GeoMX WTA. We identified more neuronal DEGs in the early tauopathy stage and a higher number of microglial DEGs at the later stage, characterized by severe tau pathology and neurodegeneration. The percentage of DEGs from different cell types reflects the development and spread of tauopathy within the neuronal population, leading to the activation of astrocytes and microglia.

The study describing the original characterization of the PS19 model reported that microglial activation precedes astrogliosis by several months, based on immunohistochemical staining^5^. However, our spatial transcriptomics characterization reveals a tight correlation between DAM and DAA signature scores, indicating the simultaneous activation of microglia and astrocytes. This simultaneous response implies that tau pathology, marked by AT8^+^ tau, initiates crosstalk between neurons and glia, as well as between astrocytes and microglia, leading to coordinated transcriptional responses in affected regions. Gene co-expression network analysis further highlights the role of complement pathway genes, such as *C1qa*, a hub gene associated with β-amyloid plaques, tau tangles, and synaptic loss, emphasizing a shared neuroinflammatory mechanism driving microglial and astrocytic activation. Synchronized activation likely contributes to neuroinflammatory feedback loops, exacerbating neuronal damage and tau pathology. The extent of DAM and DAA signatures in different brain regions is likely due to altered crosstalk resulting from microglial and astrocyte regional heterogeneity^41–42^ and subtle differences in regional tau propagation and neuronal vulnerability^43–44^. Future research should investigate the mechanistic basis of this simultaneous activation, particularly the signaling pathways linking tauopathy to shared microglial and astrocytic responses.

PGK1, a key enzyme in glycolysis, has emerged as a significant early marker of metabolic dysfunction associated with tauopathy^15–29–30–45^ and neuroinflammation^46^. Its activation has been shown to alleviate protein aggregation in various neurodegenerative conditions^30^. Moreover, a genome-wide CRISPRi-based modifier screen in iPSC-derived neurons revealed that the loss of *PGK1* reduced the tau oligomer levels in these neurons^29^. Our spatial transcriptomic analysis and RNAscope validation demonstrate *Pgk1* upregulation in the CA3 region of 2-month-old PS19 mice, preceding overt tau pathology. This early increase in *Pgk1* expression aligns with previous findings linking glycolytic activity to elevated total tau levels in CSF, underscoring its potential as an early biomarker of tau-related neurodegeneration. Mechanistically, early *Pgk1* activation in CA3 may be a compensatory response to ATP deficits or increased metabolic demand due to the accrual of p-tau in this synapse-dense hippocampal region^47^. However, the sustained upregulation of glycolytic genes in PS19 mice suggests a maladaptive state, potentially contributing to chronic metabolic stress and neuronal dysfunction. This is consistent with studies indicating that prolonged glycolytic dysfunction exacerbates tau aggregation and neuroinflammation^48–49^. Beyond its role in energy metabolism, *Pgk1* is also involved in autophagy^50^.

Glycolytic impairment may disrupt lysosomal acidification by limiting ATP availability for V-ATPase activity, in which *ATP6v1a* is critical^51–52^. Dysfunctional lysosomes, a hallmark of AD, lead to defective autophagy and the accumulation of misfolded proteins, including tau and β-amyloid. In turn, impaired lysosomal function exacerbates neuronal and glial stress, further driving disease progression. Changes in *Pgk1* expression early in the tauopathy brain may reflect an attempt to maintain ATP levels, whereas disruptions in *ATP6v1a* expression or function could indicate downstream lysosomal failure^53^. Thus, dysregulation of *Pgk1* and *ATP6v1a* represent a pathological loop linking energy deficits to lysosomal dysfunction, glial activation, and tau pathology, ultimately contributing to neurodegenerative disease progression. Understanding these mechanistic connections could open new avenues for therapeutic intervention in neurodegenerative diseases.

Integrating the spatial and temporal transcriptomic signatures in the PS19 model reveals distinct regional and stage-specific dynamics in ATP metabolic pathways. At early stages (2 months), the hippocampal regions, particularly CA3, exhibit elevated ATP metabolic processes and oxidative phosphorylation signatures, suggesting that abnormal tau levels drive an early and localized energy demand. Hyperphosphorylation of tau induced by high levels of glucose indicates the interplay between glucose metabolism and tau pathology^54^. The vulnerability of the hippocampal CA3 region^55,57^ to β-amyloid and metabolic derangements aligns with our observations of elevated ATP metabolic pathways in the CA3 region of PS19 mice. The high NTF signature scores correlate with CA3 neuronal vulnerability and are consistent with the observed transcriptional changes linked to metabolic stress and synaptic dysfunction. In contrast, cortical regions such as RSP and SS display delayed activation of similar metabolic pathways, reaching peak levels at later stages of tau pathogenesis at 6–8 months of age. This temporal progression reflects the spreading nature of tau pathology from the hippocampus to selected cortical regions in this model^58^, underscoring the importance of spatial profiling in understanding AD progression. Notably, shared region-independent signatures, such as disruptions in circadian rhythms and excitatory synaptic modulation, provide transcriptomic evidence for systemic effects of tauopathy, including sleep disturbances and memory deficits (Fig. 6C and E). This spatiotemporal analysis highlights the potential of targeting metabolic pathways, such as oxidative phosphorylation and glycolysis, at specific disease stages to mitigate tau-driven neurodegeneration. Further integration of spatial transcriptomics with proteomics and metabolomics could refine our understanding of these processes and their therapeutic implications.

## Supplementary Files

This is a list of supplementary files associated with this preprint. Click to download.

- SI2counttable.xls
- SI1figures.pdf

**Supplementary information** accompanies the manuscript on the Experimental & Molecular Medicine’s website (http://www.nature.com/emm/)

**Supplementary information** (Supplementary figures 1–5 and supplementary table 1) is available at the Experimental & Molecular Medicine’s website (http://www.nature.com/emm/)
